# Plasma Androstenedione Concentration Can Discriminate Frail versus Non-Frail Men with Prostate Cancer under Androgen Deprivation Therapy

**DOI:** 10.3390/biom13111642

**Published:** 2023-11-13

**Authors:** Mayra Alejandra Mafla-España, María Dolores Torregrosa, Manel Beamud-Cortés, Lorena Bermell-Marco, José Rubio-Briones, Omar Cauli

**Affiliations:** 1 Nursing Department, University of Valencia, 46010 Valencia, Spain; maymaes@alumni.uv.es; 2 Frailty Research Organized Group (FROG), University of Valencia, 46010 Valencia, Spain; 3 Medical Oncology Department, Doctor Peset University Hospital, 46017 Valencia, Spain; torregrosa_dol@gva.es; 4 Urology Department, Doctor Peset University Hospital, 46017 Valencia, Spain; 5 Urology Clinic, Hospital VITHAS 9 de Octubre, 46015 Valencia, Spain; 6 Chair of Healthy, Active and Participative Ageing, University of Valencia, 46010 Valencia, Spain

**Keywords:** metastatic prostate cancer, localised prostate cancer, frailty syndrome, geriatric assessment, LHRH analogues, androstenedione, testosterone, DHEA

## Abstract

Background: Androgen deprivation therapy (ADT) is a mainstay of prostate cancer in both adjuvant and palliative settings. Since androgens are crucial for functional status and psychological functions, we evaluated whether blood testosterone, androstenedione, or DHEA concentrations were associated with functional status and psychological alterations in patients with localised (PCa) or metastatic prostate cancer (mPCa) receiving ADT with analogues of luteinising hormone-releasing hormone (LHRH). Methods: The five Fried criteria were considered to identify frailty syndrome. In addition, complementary evaluations were carried out to measure other variables of interest. Sleep quality was assessed using the Athens Insomnia Scale, cognitive functions were assessed using the Mini-Mental State Examination, and symptoms of depression were measured using the Yesavage Geriatric Depression Scale. Logistic regression analysis was performed to determine if the androgens level could be related to frailty syndrome, sleep impairment, depressive symptoms, and cognitive functions. Results: The results of the multivariate analyses show that high concentrations of androstenedione were significantly associated with frailty syndrome in both groups (*p* = 0.018; odds ratio = 4.66, 95% confidence interval [1.30–16.6]). There were significant relationships between frailty syndrome and the systemic concentration of androstenedione (*p* = 0.01), but not the concentration of testosterone (*p* = 0.60) or DHEA (*p* = 0.42). In addition, the results of the non-parametric tests show significant results between a decreased gait speed in the two groups (metastatic and localised) and the concentration of androstenedione (*p* = 0.015). High androstenedione levels were associated with a slow walking speed in the mCaP group (*p* = 0.016), while high testosterone levels were associated with a better walking speed in the localised CaP group (*p* = 0.03). For the concentration of androstenedione in plasma, the area under the curve was 0.72, with a 95% CI of 0.55–0.88 with acceptable values, and with a cut-off point of 4.51 pg/mL, a sensitivity of 82.9%, and specificity of 53.8%. No relationships between the concentration of androgens in plasma and sleep quality, cognitive functions, or symptoms of depression suggest that the changes were specific to frailty syndrome. Conclusions: Further research into the role of androstenedione should be evaluated in follow-up studies in order to recommend its use as a suitable biomarker of frailty syndrome in prostate cancer patients.

## 1. Introduction

Prostate cancer (PCa) is a highly prevalent tumour type, 80–90% of cases of which are androgen-dependent at the time of initial diagnosis [1]. Testosterone is known to be a key factor in natural tumour growth; its action is regulated through the androgen receptor (AR), itself a hormone-activated transcription factor important for PCa biology and progression [2]. The treatment of this patient population focuses on the depletion of tumour-stimulating androgens, with testosterone being the major androgenic hormone [3]. Most PCas express high levels of AR, and their growth depends on testosterone produced from cholesterol both in the testis and adrenal cortex. In addition, tumour cells may develop the ability to produce testosterone to autonomously stimulate growth [4].

PCa treatment relies heavily on androgen deprivation therapy (ADT), which is used both to prevent the relapse of localised PCa and to treat mPCa. This therapy includes the use of first-line ADT agents, such as gonadotropin-releasing hormone (GnRH) agonists or GnRH antagonists [5], which aim to reduce androgen levels in the body. These treatments aim to reduce the tumour burden and improve patient survival [6]. Furthermore, ADT with LHRH analogues reduces testicular androgen production without affecting adrenal or intracrine androgen synthesis [7]. Androgens such as androstenedione are AR agonists, which may influence cancer progression. In fact, the extragonadal synthesis of androgens can produce levels in PCa tumours that exceed those found in the prostates of eugonadal men and which are sufficient to activate AR signalling [8].

In turn, given the rapid expansion of population aging [9], frailty among the elderly is emerging as one of the greatest global public health challenges for the coming century. With the increase in the number of older adults, there has also been an increase in the number of older people suffering from frailty [10]. Physiological frailty represents a state of vulnerability associated with aging and loss of the physical capacity of biological systems, along with greater associated morbidity and mortality [11]. Importantly, age-related changes in body composition play an important role in frailty [12], especially because muscle mass decreases and visceral fat mass increases with age [13]. Frailty is a dynamic state that can be influenced by different factors, including the different treatments and stages of diseases.

Of note, ADT can affect frailty with some studies, suggesting that this treatment could have a negative effect that could accelerate the physical changes experienced by older adults, including body composition [14], metabolic parameters [15,16], health and the risk of falls [17], and overall frailty levels [18,19], thereby significantly affecting patient quality of life (QoL) [20]. Furthermore, testosterone deficiency has been shown to be independently associated with frailty in older people, regardless of sex [21,22]. Moreover, the possible involvement of other androgens, such as androstenedione and dehydroepiandrosterone (DHEA), in frailty syndrome has not yet been studied in men with PCa. To date, only one long-term study performed in postmenopausal women with oestrogen receptor-positive breast cancer being treated with aromatase inhibitors has found a significant association between elevated androstenedione levels and the progression of frailty syndrome [23]. Our group previously published [24] that the presence of metastatic disease in PCa during castration-sensitive therapy with LHRH analogues is not accompanied by a general worse frailty status, e.g., the number of frailty criteria was comparable with men with localised disease under ADT to prevent cancer relapse. This means that under these conditions (ADT with LHRH analogues), frailty is not, a priori, a condition more linked to metastatic disease localised in one group, but other molecular factors are likely to be involved in frailty in both groups of patients.

The identification of blood biomarkers associated with frailty syndrome in patients with PCa may prove to be a valuable tool in identifying patients who are at risk of developing or worsening frailty syndrome. The identification of molecular biomarkers, easily detectable in blood samples, may help clinicians to classify frail patients and thereby facilitate the timely implementation of preventive and therapeutic interventions. Within the proposed role of peripheral low androgen tone linked to frailty in older men without cancer, the purpose of this current study was to assess whether testosterone, androstenedione, or DHEA levels are associated with frailty syndrome or other functional alterations in PCa during ADT and if any, can be used to classify frail versus non-frail subjects with acceptable sensitivity and specificity.

## 2. Materials and Methods

### 2.1. Design and Study Population

This study was carried out in Valencia (Spain) in collaboration with the Department of Oncological Urology at the IVO Foundation and the Oncology Service at the Doctor Peset University Hospital (both in Valencia, Spain). The study included male patients diagnosed with localised or metastatic PCa who had previously been treated by prostatectomy and radiotherapy and were receiving ADT using LHRH analogues, and who spoke the Spanish language and could understand the content of the questions posed in the psychological and functional evaluations. In total, 65 men agreed to participate in the study and provided their written informed consent by signing a form.

### 2.2. Assessment of Frailty Syndrome

The physical frailty phenotype proposed by Fried et al. was used to assess frailty syndrome in this work. These criteria assess (1) unintentional weight loss of 4.5 kg or more in the year prior; (2) self-reported exhaustion when the participants answered ‘always’ or ‘often’ to the question “How often in the last week did you feel that everything was an effort?”; (3) decreased physical activity, with the total amount of time expended on physical activity per week (in minutes) being in the lowest quintile on the International Physical Activity Questionnaire; (4) slow walking speed, with participants in the lowest quintile for a height-adjusted (by 7 s for heights ≥ 1.73 m and 6 s for heights < 1.73 m) gait time of 4.6 m being considered frail; and (5) muscle weakness measured using a digital hand dynamometer to determine the grip strength three times in each hand, with those in the last quintile being considered frail. Accordingly, individuals who met one or two of these criteria were classified as pre-frail, those who met three or more were classified as frail, and participants who did not meet any of the criteria were classified as robust.

### 2.3. Geriatric Assessment

The Mini-Mental State Examination (MMSE) is a commonly used tool for assessing cognitive function and detecting dementia. This test can be performed by professionals with minimal training and takes about 10 min to complete. The Mini Examen Cognoscitivo (MEC) is the accepted and validated Spanish version of the MMSE [25]. There are two versions of the MMSE containing 30 and 35 questions, respectively, with the 30-point version being more useful for international comparisons. Its items explore 5 cognitive areas: orientation (temporal and spatial), fixation, attention and calculation, memory, and language. Higher scores indicate better cognitive function, while scores < 23 points are often used as a cut-off for detecting cognitive decline in older people.

The Athens Insomnia Scale (AIS) is a psychometric instrument that measures sleep disturbance. This tool comprises 8 questions answered on a scale of 0 to 3, where the first 5 questions are related to night-time sleep (difficulty initiating sleep, difficulty maintaining sleep, and waking up early in the morning), while the 3 remaining questions address daytime dysfunction because of any reported sleep disturbance. This includes the subjective assessment of the participant regarding their sense of wellbeing, ability to function, and daytime sleepiness. The total score ranges from 0 to 24 points and higher scores indicate a greater severity of the sleep problem, with the cut-off point being 6 points [26].

The Abbreviated Geriatric Depression Scale (GDS) is a questionnaire used to screen depression in people aged over 65 years. The GDS-15 has a dichotomous affirmative or negative response pattern for 15 items that investigate only cognitive disorder symptoms resulting from a major depressive episode in the 15 days prior. The GDS-15 excludes somatic symptoms of depression, such as sleep difficulties, appetite disturbances, lack of concentration, and fatigue, among others. The score on this scale ranges from 0 to 15 points with a cut-off of 5 points or more, indicating the presence of symptoms of depression. Finally, the Cumulative Rating Scale of Geriatric Illnesses (CIRS-G) was used to assess the comorbidity index [27].

### 2.4. Measurement of Hormones Present in Blood Plasma

Blood samples were collected between 2:00 and 3:00 p.m. and were then centrifuged at room temperature at 1500 rpm for 5 min. The supernatant was stored in 1 mL aliquots in Eppendorf tubes and frozen at −20 °C until further analysis. Testosterone concentrations were measured by chemiluminescent microparticle immunoassay (CMIA). Dihydrotestosterone and androstenedione concentrations were measured using an ELISA kit (Cloud-Clone Corp., Houston, TX, USA, ref. CEA398Ge and Arbor assays ref. K070-H5, respectively), following the manufacturer’s instructions.

### 2.5. Statistical Analysis

Various statistical analysis techniques were used to study the data. For the qualitative variables, the frequency distribution and corresponding percentages were calculated. In turn, measures of central tendency, such as the arithmetic mean, and measures of dispersion, such as the standard deviation (SD) and range of values, were obtained for the quantitative variables. Non-parametric Spearman correlation tests were used to analyse the correlations among the quantitative variables. Patients were classified into two groups: robust (without frailty criteria) and pre-frail/frail (meeting at least one frailty criterion). The non-parametric Mann–Whitney U and Kruskal–Wallis tests were used for bivariate analysis between these groups. Logistic regression analysis was subsequently performed, with the aim of creating a predictive model to determine the associations with the variables identified in the bivariate analyses. Logistic regression allows for the simultaneous evaluation of several factors that may or may not be related to the dependent variable (the presence or absence of frailty criteria). To measure the discriminatory accuracy of the predictive model in terms of biomarkers between robust and pre-frail/frail patients, the C statistic, also known as the area under the receiver operating characteristic curve (AUC), was used. A confidence level of 95% was established for all the statistical tests, and a score (*p*) less than 0.05 was considered significant. All the data were analysed using SPSS software (version 24, IBM Corp., Armonk, NY, USA).

## 3. Results

### 3.1. Sociodemographic and Clinical Data

Sixty-five men diagnosed with PCa or mPCa, with a mean age of 73.7 ± 1.11 years (SEM; range: 55–92 years), were included in the study. They were all patients living in Valencia (Spain) and were married (86.2%), divorced (4.6%), separated (6.2%), or widowed (3.1%). The mean duration of androgen blockade was 71.56 ± 8.59 (SEM; range: 1–211 months). At the time of the study, 63% of the patients had undergone a prostatectomy, and 37% had not. The mean BMI of the participants was 27.7 ± 0.42 (SEM; range: 20–38), and according to these data, 23.1% of the patients had a normal weight (BMI = 18.5–24.9 kg/m^2^), 55.4% were overweight (BMI = 25–29.9 kg/m^2^), and 21.5% were obese (BMI > 30 kg/m^2^). None of the patients were underweight (BMI < 18.5 kg/m^2^). Approximately 58.5% of the patients had central or android obesity, with a waist circumference ≥ 102 cm. The age-adjusted Charlson index indicated a mean index of 2.87 ± 0.20 (SEM; range: 0–7), as shown in Table 1.

### 3.2. Relationship between Frailty Syndrome and Androgen Concentration in Metastatic and Localised Prostate Cancer

**Figure 1:**
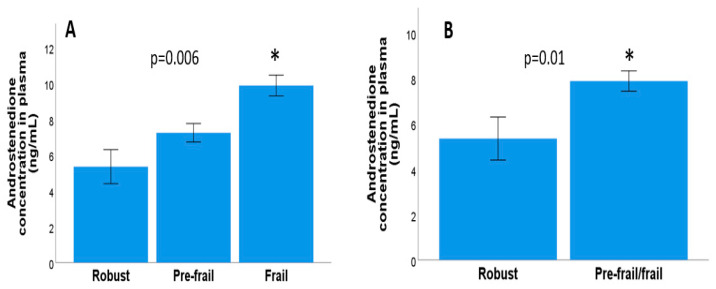
(**A**) Plasma androstenedione concentration in the three frailty groups in men with metastatic and localised prostate cancer. (**B**) Plasma androstenedione concentration and dichotomised frailty (robust/prefrail-frail) in men with metastatic and localised prostate cancer. * means significant differences compared with robust individuals.

**Figure 2:**
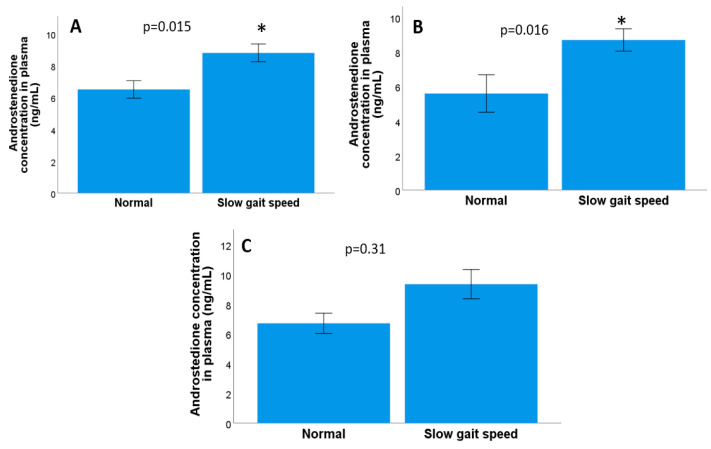
(**A**). Plasma androstenedione concentration and gait speed in men with localised and metastatic prostate cancer. (**B**) Plasma androstenedione concentration and gait speed in men with metastatic prostate cancer. (**C**) Plasma androstenedione concentration and gait speed in men with localised prostate cancer. * means significant differences compared with normal weight speed.

No significant correlations were found between the number of frailty criteria and the plasma testosterone concentration (Rho = −0.07, *p* = 0.48, non-parametric Kendall Tau *b* test) or between the plasma testosterone concentration and frailty levels in either three categories (*p* = 0.47, Kruskal–Wallis test) or two categories (robust and pre-frail/frail; *p* = 0.60, Mann–Whitney U test). Furthermore, there were no significant differences between the testosterone concentration and each of the frailty criteria evaluated. Moreover, both involuntary weight loss (*p* = 0.80) and fatigue (*p* = 0.71) were linked to decreased physical activity (*p* = 0.11), muscle strength (*p* = 0.62), and gait speed (*p* = 0.31, Mann–Whitney U test in all cases). However, significant correlations were found between the number of frailty criteria and the plasma androstenedione concentration (Rho = 0.30, *p* = 0.003, Kendall Tau *b* correlation). In addition, significant differences were also observed in the three categories of frailty levels and the concentration of plasma androstenedione (*p* = 0.006, Kruskal–Wallis test), as shown in Figure 1A, and between the two categories of frailty (robust and pre-fragile/fragile) and the plasma androstenedione concentration (*p* = 0.01, Mann–Whitney–U test), as shown in Figure 1B. We also evaluated any potential links between each of the frailty criteria and the plasma androstenedione concentration. There were no significant differences between involuntary weight loss, fatigue, physical activity, or decreased muscle strength and the plasma androstenedione concentration (*p* = 0.31, *p* = 0.45, *p* = 0.11, *p* = 0.11, *p* = 0.29), but there were differences between a decreased gait speed and the plasma androstenedione concentration (*p* = 0.015, Mann–Whitney U test), as shown in Figure 2A.

No significant correlations were found between the number of frailty criteria and the DHEA concentration (Rho = −0.05, *p* = 0.62, Kendall Tau *b* correlation). Likewise, no significant differences were found between frailty categorised according to the DHEA concentration (*p* = 0.60, Kruskal–Wallis test) or with the dichotomous frailty variable (*p* = 0.42, Mann–Whitney U test). In addition, there were no significant differences between the frailty criteria and the DHEA concentration. In the individual analysis, Mann–Whitney–U tests showed that there were no statistically significant differences between DHEA levels and involuntary weight loss (*p* = 71) fatigue (*p* = 0.88), decreased physical activity (*p* = 0.44), decreased muscle strength (*p* = 0.19), or a slow walking speed (*p* = 1.00).

In addition, correlations were made with the purpose of identifying possible relationships between the concentration of androstenedione and the testosterone and DHEA hormones. The results obtained reveal that no significant correlations were observed between androstenedione and the hormones (Rho = 0.08, *p* = 0.53, Spearman’s correlation; Rho = −0.03, *p* = 0.88, Spearman’s correlation, respectively). In contrast, there was a significant inverse correlation between the plasma DHEA and testosterone concentrations (rho = −0.42, *p* = 0.002).

### 3.3. Relationship between Frailty Syndrome and Androgen Concentration in Metastatic Prostate Cancer

Our results show that there were no significant correlations between the number of frailty criteria and the testosterone concentration in patients with mPCa (Rho = 0.23, *p* = 0.15, Kendall Tau *b* correlation). Furthermore, the results of the non-parametric tests show no significant differences between the categorical frailty variable and the testosterone concentration (*p* = 0.28, Kruskal–Wallis test) or the dichotomous frailty variable (*p* = 0.40, Mann–Whitney U test). In addition, there were no significant differences between the testosterone concentration and the five frailty criteria: involuntary weight loss (*p* = 0.52), fatigue (*p* = 0.17), decreased physical activity (*p* = 0.49), decreased muscle strength (*p* = 0.40), and slow walking speed (*p* = 0.36, Mann–Whitney U test in all cases).

Significant correlations were found between the number of frailty criteria and the androstenedione concentration (Rho = 0.34, *p* = 0.02, Kendall Tau *b* correlation). However, no significant differences were found between the categorical frailty variable and the androstenedione concentration (*p* = 0.08, Kruskal–Wallis test) or the dichotomous frailty variable (*p* = 0.14, Mann–Whitney U test). Likewise, no significant differences were observed in four of the frailty criteria: involuntary weight loss (*p* = 0.71), fatigue (*p* = 0.74), decreased physical activity (*p* = 0.45), and decreased muscle strength (*p* = 0.17), but there were significant differences between the walking speed and the androstenedione concentration (*p* = 0.016, Mann–Whitney U test in all cases), as shown in Figure 2B.

No significant correlations were found between the number of frailty criteria and the DHEA concentration (Rho = −0.04, *p* = 0.75, Kendall Tau *b* correlation). Likewise, no significant differences were observed with the categorised (*p* = 0.15, Kruskal–Wallis test) or dichotomous (*p* = 0.97, Mann–Whitney U test) frailty variables. In addition, no significant differences were found between the DHEA concentration and the five frailty criteria: unintentional weight loss (*p* = 0.44), fatigue (*p* = 0.34), decreased physical activity (*p* = 0.23), decreased muscle strength (*p* = 0.06), and slow gait (*p* = 0.72, Mann–Whitney U test in all cases).

### 3.4. Relationship between Frailty Syndrome and Androgen Concentration in Localised Prostrate Cancer

No significant correlations were found between the number of frailty criteria and the testosterone concentration in patients with localised PCa (Rho = −0.25, *p* = 0.05, Kendall Tau *b* correlation). Nor were significant differences observed in relation to the categorised (*p* = 0.39, Kruskal–Wallis test) or dichotomous frailty variables (*p* = 0.07, Mann–Whitney U test) or with involuntary weight loss (*p* = 0.88), fatigue (*p* = 0.15), decreased physical activity (*p* = 0.35), or decreased muscle strength (*p* = 1.00), although significant differences were found between the walking speed and the testosterone concentration (*p* = 0.03, Mann–Whitney U test in all cases).

No significant correlation was found between the number of frailty criteria and the androstenedione concentration (Rho = 0.27, *p* = 0.05, Kendall Tau *b* correlation). Likewise, no significant differences were observed between the categorised (*p* = 0.07, Kruskal–Wallis test), as shown in Figure 1C or dichotomous (*p* = 0.08, Mann–Whitney U test). Variables of frailty or with the five frailty criteria: involuntary weight loss (*p* = 0.30), fatigue (*p* = 0.17), decreased physical activity (*p* = 0.14), decreased muscle strength (*p* = 0.96), and slow walking speed (*p* = 0.31, Mann–Whitney U test in all cases), as shown in Figure 2C.

Similarly, no significant correlations were found between the number of frailty criteria and the DHEA concentration (Rho = −0.04, *p* = 0.74, Kendall Tau *b* correlation). There were no significant differences observed in relation to the categorised (*p* = 0.24, Kruskal–Wallis test) or dichotomous (*p* = 0.25, Mann–Whitney U test) variables of frailty or with the five frailty criteria: involuntary weight loss (*p* = 0.40), fatigue (*p* = 0.83), decreased physical activity (*p* = 0.43), decreased muscle strength (*p* = 0.42), and slow walking speed (*p* = 0.39, Mann–Whitney U test in all cases).

### 3.5. Relationship between Androgen Concentration and the Geriatric Assessment Criteria in Metastatic and Localised Prostate Cancer

Spearman tests found no significant correlations were found between the testosterone concentration and each of the MMSE subscales: time orientation (Rho = 0.07, *p* = 0.58), fixation (Rho = −0.18, *p* = 0.13), delayed recall (Rho = −0.04, *p* = 0.74), language (Rho = 0.20, *p* = 0.10), and the total MMSE score (Rho = 0.21, *p* = 0.09). However, there were significant correlations with the other two subscales: spatial orientation (Rho = 0.35, *p* = 0.004), and attention and calculation (Rho = 0.34, *p* = 0.005). Finally, no significant correlations were found between the presence of insomnia and the testosterone concentration (Rho = −0.05, *p* = 0.64) or with symptoms of depression (Rho = −0.15, *p* = 0.22).

In addition, Spearman tests found no significant correlations between the MMSE subscales and the androstenedione concentration. Subscales that did not show a significant correlation were temporal orientation (Rho = −0.3, *p* = 0.83), partial orientation (Rho = −0.009, *p* = 0.94), fixation (Rho = −0.15, *p* = 0.28), attention and calculation (Rho = 0.06, *p* = 0.64), delayed recall (Rho = −0.14, *p* = 0.32), language (Rho = 0.12, *p* = 0.34), and overall MMSE score (Rho = −0.02, *p* = 0.89). In addition to the MMSE subscales, there was also no significant correlation between the insomnia and the plasma androstenedione concentrations (Rho = 0.13, *p* = 0.33). Similarly, there was no significant correlation between the presence of symptoms of depression and the plasma androstenedione concentration (Rho = −0.07, *p* = 0.63).

Also using Spearman tests, no significant correlations were found between the DHEA concentration and any of the MMSE subscales: temporal orientation (Rho = −0.73, *p* = 0.60), spatial orientation (Rho = −0.14, *p* = 0.30), fixation (Rho = −0.04, *p* = 0.72), attention and calculation (Rho = −0.15, *p* = 0.26), delayed recall (Rho = 0.23, *p* = 0.08), language (Rho = 0.13, *p* = 0.33), and the overall MMSE score (Rho = 0.06, *p* = 0.64). Likewise, no significant correlations were found between the presence of insomnia and the concentration of DHEA (Rho = 0.05, *p* = 0.69) or symptoms of depression (Rho = 0.16, *p* = 0.22). In summary, the findings suggest that plasma androstenedione and DHEA levels were not related to the geriatric assessment outcomes in men with localised and metastatic PCa.

### 3.6. Fragility Syndrome and Plasma Androstenedione Concentration

**Figure 3:**
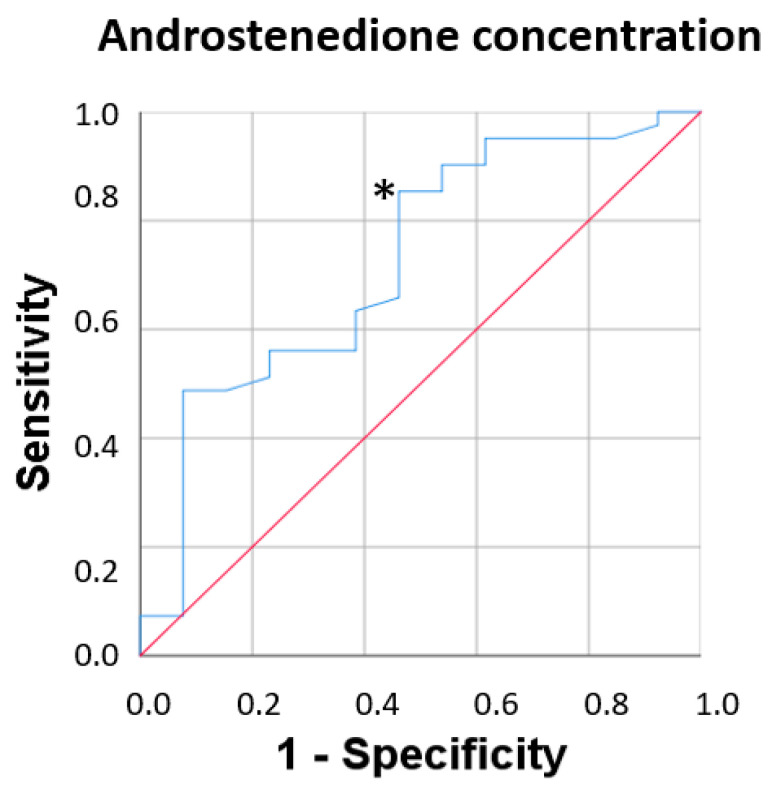
Receiver operating characteristic (ROC) curve for androstenedione. The red line represents a hypothetical ROC curve of a perfect classifier. The blue curve represents the actual ROC curve classifier. * indicates the point selected as a cut-off with the best sensitivity and specificity.

Logistic regression analysis was used to determine the associations with the significant variables identified in the bivariate analyses. As a dependent variable, frailty was divided into two categories (robust = 0 and prefrail/fragile = 1). Significant associations were found between frailty and the plasma concentration of androstenedione (*p* = 0.018; odds ratio [OR] = 4.66, 95% CI [1.30, 16.6]) but no such associations were identified between frailty and testosterone (*p* = 0.36; OR = 0.89, 95% CI [0.69, 1.14]) and DHEA (*p* = 0.23; OR = 0.97, 95% CI [0.92, 1.01], as shown in Figure 3.

Next, we performed a receiver operating characteristic curve analysis to assess the diagnostic power of this biomarker to detect frailty syndrome when categorised as robust and prefrail/frail in men who met at least one frailty criterion. This analysis provided information on the relationship between the sensitivity and specificity of plasma androstenedione concentrations for the diagnosis of frailty. For the concentration of androstenedione in plasma, the area under the curve was 0.72, with a 95% CI of 0.55–0.88 with acceptable values and a cut-off point of 4.51, sensitivity of 82.9%, and specificity of 53.8%.

## 4. Discussion

This study, performed in a population of men with metastatic and localised PCa who were receiving therapy with LHRH analogues, showed, for the first time, significant associations between frailty syndrome and the androstenedione concentration, but not with other androgens, e.g., testosterone or DHEA concentrations. Patients with mPCa and localised PCa who were frail (met three or more frailty criteria) had high levels of androstenedione in plasma. Androstenedione is an androgen produced primarily in the adrenal glands and testes in men. Under normal conditions, adrenal glands produce 2–3 mg/day and the testes around 0.5 mg/day [28]. The importance of androstenedione besides stimulating androgen receptors, is related to the ability of the human body to convert it into other hormones, such as testosterone and oestrogen [29].

It Is important to understand the interplay between these hormones in the context of pCa and its treatment with LHRH analogues. pCa is sensitive to the action of male sex hormones, especially testosterone and its metabolites. These hormones stimulate the growth and proliferation of malignant prostate cells [2]. LHRH analogues are used as a means of ADT in pCa. These analogues reduce circulating testosterone levels by blocking LHRH production in the hypothalamus, which in turn decreases testosterone production in the testicles [6,30]. Although LHRH analogues reduce testosterone levels, they do not directly affect androstenedione production in the adrenal glands. As a result, patients with pCa may have higher androstenedione levels despite receiving treatment with LHRH analogues. The reduction of testosterone by androgen deprivation therapy has similar mechanisms with DHEA, but not with androstenedione, thus suggesting that the increase in androstenedione associated with frailty is due to synthesis mechanisms in other tissues that are not regulated through the hypothalamic gonadal axis. Importantly, endocrine tissues, such as the gonads and adrenal glands, can produce active steroid hormones from cholesterol [31]. While in other tissues, steroid synthesis relies primarily on the conversion of various circulating precursors, adipose tissue, in addition to its function as a relevant site of steroid conversion, possesses the ability to initiate steroid production de novo [32,33]. In this context, the aromatase enzyme emerges as an essential component in the biosynthesis of sex hormones. The influence of aromatase activity is closely linked to the local availability of its androgenic substrates. In adipose tissue, where it plays a prominent role, androstenedione appears as the main substrate for aromatase. Androstenedione, provided by dehydroepiandrosterone (DHEA) and its sulphated form, both generated in the adrenal glands, is subjected to aromatisation, leading to the synthesis of estrone [34]. Consequently, the premise is raised that the inhibition of aromatase activity in patients could trigger an increase in androstenedione production. The results of a longitudinal study, carried out in patients with breast cancer undergoing hormonal treatment with aromatase inhibitors, support this idea [23]. There was a significant increase in the plasma levels of androstenedione due to the inhibition of aromatase activity, which highlights the importance of this enzyme in the regulation of hormone synthesis. However, additional investigations are required to establish with certainty the origin of elevated androstenedione concentrations in frail patients. Intratumorally steroidogenesis is involved in the production of dihydrotestosterone (DHT) from testosterone. Steroids formed in the steroidogenesis pathway include pregnenolone, DHEA, progesterone, and androstenedione [35]. Intratumoral steroidogenesis is one of the mechanisms by which cancer cells can evade ADT and continue their growth and development. By producing their own hormones within the tumour, cancer cells can maintain their activity and proliferation [36]; however, we observed an increase also in patients with localised PCa, so this possibility seems unlikely in these patients. An analysis of fat tissue in frail PCa patients will reveal its role in the increase in the production of androstenedione in frail patients.

The results of this study on the individual frailty syndrome components according to the physical frailty phenotype show significant differences between the gait speed and the androstenedione concentrations in patients with mPCa. Of note, participants with high androstenedione levels had a slow walking speed, while significant differences were observed between the gait speed and the testosterone concentration among those with localised PCa. In other words, participants with high testosterone levels had a better gait speed. Gait speed depends on both physical and neural factors, which are both altered during aging, and so it is conceivable that decreased androgen production while receiving LHRH analogue therapy may impact neuromuscular unit performance. Indeed, as they age, men are more prone to deteriorating physical function, which ties in with this previous idea.

In addition to identifying the androstenedione concentration as a possible biomarker to detect frail patients, based on the present study design, it was not possible to assess whether the concentration of the hormone in frail PCa patients also occurred in frail men without PCa. By comparing the data reported in the literature for plasma androstenedione concentration in older men without cancer, we found plasma concentrations ranging from 0.2 to 1.57 ng/mL [37,38], which are lower compared to the concentrations found in our study in PCa patients (range of 1.72–12.04 ng/mL, mean value of 7.27 ng/mL). Future studies run in parallel with two control groups, e.g., men with PCa without ADT and men without PCa, would elucidate the specificity of these associations between frailty and the plasma androstenedione concentration.

It is well established that ADT can cause significant morbidity because of toxicities that reduce QoL [39], and that this is associated with serious adverse effects, such as sarcopenia and muscle weakness, which closely overlap with the physical changes associated with frailty syndrome [15,16]. Sarcopenia is a syndrome characterised by the progressive and generalised loss of skeletal muscle mass and strength, and is mainly associated with aging [40]. However, many conditions other than aging, such as chronic disease, immobilisation, malnutrition, and anabolic hormone deficiency (e.g., sex hormones) may be involved in the aetiology of sarcopenia [41]. Indeed, androgens are important determinants of body composition in men and play an important role in frailty syndrome [21]. Low testosterone levels in men are associated with reduced skeletal muscle mass and strength [42].

While the correlations and causal relationships between sex hormones and muscle mass and function have not yet been fully elucidated, both of these factors are known to decline with age [43]. Sarcopenia is a condition that arises because of systemic inflammation, which is commonly observed in cases of malignancy. As part of the systemic inflammatory response generated by tumours, proinflammatory cytokines have a strong catabolic effect on the host metabolism, leading to muscle failure [44]. Furthermore, recent studies have indicated that chronic inflammation contributes to sarcopenia and that systemic inflammatory mediators affect muscle protein metabolism during aging [45]. Also of note, high levels of certain inflammatory markers in an elderly population have been reported to be significantly associated with determining sarcopenia status, including muscle mass index, gait speed, and grip strength [46].

The concentration of androgens in PCa patients under ADT with LHRH analogues was not associated with other clinical variables, such as sleep quality, symptoms of depression, cognitive function, and testosterone, androstenedione, and DHEA. Thus, since PCa largely affects older men, it is important to evaluate, in future studies on biomarkers, the typical age-related physiological changes when treating older men with PCa [47]. Patients with PCa may have a wide range of follow-up needs, including management of the physical and psychosocial side effects of diagnosis and treatment [48]. Carrying out a bio-psycho-social assessment in cancer patients is crucial because this disease not only affects the body physically but also significantly impacts the emotional, mental, and social well-being of the individual.

The updated American Society of Clinical Oncology guidance recommends that all cancer patients aged 65 years or older who are receiving systemic therapy and with impairments identified by a geriatric assessment (GA), which includes physical and cognitive function, emotional health, nutrition, comorbid conditions, polypharmacy, and social support, should have GA-guided management (GAM) included in their care plan. GAM includes the use of GA results to (1) inform decision-making about cancer treatment, and (2) address gaps through appropriate interventions, counselling, and/or referrals [49]. The use of GAM is vital to reduce toxicity and improve treatment adherence [50,51], and our study suggests that the evaluation of biomarkers associated with physical and psychological well-being are also necessary. The information related to objective measurements, such as blood biomarkers in this field, would help clinicians to avoid both the over-treatment of frail patients and the under-treatment of robust patients and to share clinical decision with patients and their families [49].

As a limitation of this study, we cannot affirm increases in androstenedione as a mechanism of ADT since we did not have a group of patients without ADT as it was beyond the scope of the study. A pioneer study [52] on castrated men with metastatic PCa showed a reduction in plasma androstenedione concentrations, reaching 1.1 ng/mL. In the case of ADT in our study, we detected concentrations of androstenedione ranging from 1.72 to 12.04 ng/mL (mean value of 7.27 ng/mL), so it seems that under ADT (chemical castration), the level of hormone is higher than in surgical castration. In addition, the effect of ADT on androstenedione in frail PCa patients also seem similar to that in frail women with localised breast cancer, as it was previously reported [23] that frail breast cancer women receiving aromatase inhibitors to lower their estrogen level also showed an increase in the androstenedione concentration compared to non-frail women. In both genders, androstenedione can be also released from other tissues, such as fat tissue or adrenal glands, and this issue need to be further investigated in the future also by comparing prostate cancer and breast cancer patients without any hormonal treatment.

## 5. Conclusions

In this current work, significant associations were found between frailty syndrome and the androstenedione concentration in patients with mPCa and localised PCa. The mPCa group had higher levels of androstenedione, and this was associated with a decreased gait speed. This suggests that androstenedione levels may be related to the presence or severity of frailty syndrome in this population. In addition, participants with localised PCa who had high testosterone levels had a better walking speed. All these findings provide important information about the relationship between hormone levels (androstenedione and testosterone) and physical function (gait speed) in patients with localised and metastatic PCa receiving ADT with LHRH analogue therapy. Importantly, these results may help guide future research and therapeutic strategies to improve the quality of life and functionality of these patients. However, further studies with larger sample sizes and long-term follow-up are recommended to validate these findings and to infer about a causal relationship.

## Figures and Tables

**Table 1:** Sociodemographic and clinical data between frailty syndrome and androgen concentration in metastatic and localised prostate cancer.

Variables	Percentage Frequency for Categorical Variables or Mean ± Standard Error of the Mean (Range Min–Max) for Discrete Variables	Patients with Metastatic Prostate Cancer (*n* = 29)	Patients with Localised Prostate Cancer (*n* = 36)
Age	73.7 ± 1.11 (55–92)	74.6 ± 1.5	72.9 ± 1.6
Previous prostatectomy			
Yes	38 (61.9%)	Yes (58.6%)	Yes (66.7%)
No	25 (38.1%)	No (41.4%)	No (33.3%)
BMI (kg/m^2^)	0		
Underweight (<18.5)			
Normal (18.5–24.9)	15 (23.1%)	9 (31%)	6 (16.7%)
Overweight (25–29.9)	36 (55.4%)	16 (55.2%)	20 (55.6%)
Obese (>30)	14 (21.5%)	4 (13.7%)	10 (27.8%)
Gleason Index	7.30 ± 0.14 (5–10)	7.5 ± 0.22	7.1 ± 0.17
Charlson Comorbidity Index	2.87 ± 0.20 (0–7)	2.6 ± 0.39	3.3 ± 0.23
Number of Fried criteria	1.46 ± 0.13 (0–4)	1.55 ± 0.20 (0–4)	1.38 ± 0.18 (0–4)
Frailty syndrome criteria:			
Robust (0 criteria)	16 (24.6%)	6 (20.7%)	10 (27.8%)
Pre-frail (1–2 criteria)	38 (58.5%)	17 (58.6%)	21 (58.3%)
Frail (>3 criteria)	11 (16.9%)	6 (20.7%)	5 (13.9)

## Data Availability

The raw data supporting the results of this paper will be made accessible upon request by the corresponding author for scientific purposes.
